# Immunoglobulin directly enhances differentiation of oligodendrocyte-precursor cells and remyelination

**DOI:** 10.1038/s41598-023-36532-3

**Published:** 2023-06-09

**Authors:** Yaguang Li, Daisuke Noto, Yasunobu Hoshino, Miho Mizuno, Soichiro Yoshikawa, Sachiko Miyake

**Affiliations:** 1 Department of Immunology, Juntendo University School of Medicine, 2-1-1 Hongo, Bunkyo-ku, Tokyo, 113-8421 Japan; 2 Department of Neurology, Juntendo University School of Medicine, Tokyo, Japan

**Keywords:** Neuroimmunology, Oligodendrocyte

## Abstract

Multiple sclerosis (MS) is an inflammatory demyelinating disease characterized by multiple lesions in the central nervous system. Although the role of B cells in MS pathogenesis has attracted much attention, but the detailed mechanisms remain unclear. To investigate the effects of B cells on demyelination, we analyzed a cuprizone-induced demyelination model, and found that demyelination was significantly exacerbated in B cell-deficient mice. We next investigated whether immunoglobulin affected the myelin formation process using organotypic brain slice cultures and revealed that remyelination was improved in immunoglobulin-treated groups compared with the control group. Analysis of oligodendrocyte-precursor cell (OPC) monocultures showed that immunoglobulins directly affected on OPCs and promoted their differentiation and myelination. Furthermore, OPCs expressed FcγRI and FcγRIII, two receptors that were revealed to mediate the effects of IgG. To the best of our knowledge, this is the first study to demonstrate that B cells act in an inhibitory manner against cuprizone-induced demyelination, while immunoglobulins enhance remyelination following demyelination. Analysis of the culture system revealed that immunoglobulins directly act on OPCs to promote their differentiation and myelination. Future studies to elucidate the effects of immunoglobulins on OPCs in vivo and the detailed mechanisms of these effects may lead to new treatments for demyelinating diseases.

## Introduction

Multiple sclerosis (MS) is an inflammatory demyelinating disease characterized by multiple demyelinating lesions with lymphocytic infiltration, antibody deposition, and complement activation in the central nervous system (CNS), as well as a variety of neurological symptoms^1^. Genome-wide association studies of MS revealed that many disease susceptibility genes encode molecules involved in the differentiation, activation, and proliferation of CD4^+^ helper T cells, suggesting a strong involvement of T cell-mediated autoimmune mechanisms in the pathogenesis of MS^2–5^. A role for T cells in MS pathogenesis is also supported by the fact that anti-very-late-antigen-4 antibodies, which inhibit T cell entry into the CNS, very effectively reduce relapse in patients with relapse-remitting MS (RRMS)^6^. However, secondary progressive MS (SPMS), which shows a progressive disease course characterized by the accumulation of irreversible neurological disabilities independent of relapses, does not respond to T cell-targeted therapy and causes impaired remyelination and neurodegeneration in demyelinating foci, implicating a different pathogenesis^7,8^. However, the mechanism of impaired remyelination remains unclear, and its elucidation may lead to the development of new therapies for MS, especially SPMS, for which there are currently few effective treatments.

The role of B cells in the pathogenesis of MS remains unclear. The presence of oligoclonal bands (OCB) in cerebrospinal fluid (CSF) is one of the hallmarks of MS, and clonal proliferation and somatic mutations of B cells in CSF have been reported, suggesting a B cell response to CNS antigen^9–11^. However, the roles of these B cells and antibodies in the pathogenesis of MS have not been clearly elucidated. Antibodies comprising OCB can reportedly recognize a variety of intracellular antigens, suggesting that they recognize cell debris^12^. In addition, autoantibodies recognizing various myelin-derived antigens have been reported in MS, but there are large differences among reports and individuals, suggesting that these autoantigens may be produced secondarily by tissue destruction. Recently, the efficacy of anti-CD20 antibody therapy for patients with RRMS renewed attention to B cells in the pathogenesis of this disease. Because the effects of anti-CD20 antibodies appear before serum levels of IgM and IgG decrease^13^, B cell functions outside of antibody production (e.g., antigen presentation to T cells and cytokine production) are thought to be related to MS pathology. In SPMS, ectopic lymphoid tissue with germinal centers is formed in the meninges, and B cells produce immunoglobulins intrathecally. However, the relationship between SPMS pathogenesis and these geminal centers or intrathecal antibody production has not yet been clarified. Moreover, B cell infiltration of the meninges reportedly correlates with cortical lesions^14,15^, and B cell-derived humoral factors adversely affect oligodendrocytes and neurons^16,17^. However, previous reports suggest that B cells and immunoglobulin play facilitative roles in myelination during mouse development^18^, and B cell infiltration of the meninges acts in an immunoregulatory manner^19^. Accordingly, the role of B cells and immunoglobulin in remyelination after pathological demyelination remains largely unclear.

In this study, we found that demyelination is exacerbated in a cuprizone-induced demyelination model established in B cell-deficient mice. Using an organotypic brain slice culture system, we reveal that immunoglobulin treatment promoted remyelination. Furthermore, we show that immunoglobulin promoted the differentiation and myelination of cultured oligodendrocyte-precursor cells (OPCs), and immunoglobulin G-affected OPCs via both FcγRI and FcγRIII. These results shed light on the protective role of immunoglobulins in remyelination through their facilitative role on the development of OPCs, which may lead to the development of new treatments for demyelinating diseases.

## Material and methods

### Animals

For organotypic brain slice culture and mixed glial culture, pregnant female C57BL/6J (B6) mice at embryonic day 14 were purchased from CLEA Laboratory Animal Corporation (Tokyo, Japan). muMT mice, which lacked mature B cells, were designed and purchased from Jackson Laboratory (Bar Harbor, ME, USA) for use in in vivo demyelination studies. All mice were well-bred under specific pathogen-free conditions and sacrificed under isoflurane anesthesia. This research was approved by the Juntendo University Graduate School of Medicine Animal Experimental Committee (Permit Number: 2022054). All methods were carried out in accordance with relevant guidelines and regulations and are reported in accordance with ARRIVE guidelines.

### In vivo demyelination model and tissue assay

Six-week-old muMT male mice were fed a chow diet including 0.2% cuprizone (bis-cyclohexanone oxaldihydrazone; Sigma, St. Louis, MO, USA) for 3 weeks to induce the acute phase of demyelination, while control mice were fed standard chow. Mice were subsequently anesthetized with isoflurane and transcranially perfused with 4% paraformaldehyde (PFA) overnight. After removing and dehydrating brain in 5%, 10%, and 20% sucrose solutions in phosphate-buffered saline (PBS). Brains were finally embedded with Optimal Temperature Cutting compound (Sakura Finetek, Tokyo, Japan). For immunofluorescence staining, brains were cut at a 14 μm thickness and blocked with 10% donkey serum (Jackson ImmunoResearch, West Grove, PA, USA) for 1 h at room temperature. Rat anti-mouse myelin basic protein (MBP) antibody (Merck, Darmstadt, Germany, MAB386, 1:500) was used as the primary antibody at 4 °C overnight. Alexa Fluor 488-conjugated donkey anti-rat IgG (Jackson ImmunoResearch, 712–545-153, 1:100) was used as the secondary antibody for 1 h at room temperature. Images were captured with a BZ-X700 fluorescence microscope (Keyence, Tokyo, Japan) and myelinated areas were measured with ImageJ software (National Institutes of Health, Bethesda, MD, USA).

### Organotypic brain slice culture and induction of demyelination

Cerebellums were collected from postnatal day 9–10 (P9–10) B6 mice after decapitation and sagittally sliced at a 300 μm thickness with a McIlwain tissue chopper (Mickle Laboratory Engineering, Guildford, UK). Porous translucent membrane inserts (Millicell-CM: PICM03050, Millipore, Billerica, MA, USA) were prepared in six-well culture plates. Cerebellum tissue slices were transferred onto the insert at a density of 4–5 pieces per well, cultured for 7 d in slice culture medium consisting of 49% Opti-MEM (Thermo Fisher Scientific, Waltham, MA, USA), 25% Hanks’ Balanced Salt Solution (Thermo Fisher Scientific), 25% heat-inactivated horse serum, 5 mg/mL D-glucose (Wako Chemicals, Osaka, Japan), and 1% penicillin/streptomycin (Thermo Fisher Scientific). Demyelination was induced with 0.5 mg/mL lysolecithin (LPC). Slices were washed after 24 h incubation and cultured in standard slice culture medium for another 6 d. Tissue slices were permeabilized and blocked with PBS containing 1% Triton X (Wako Chemicals), 2% bovine serum albumin (Iwai Chemicals, Tokyo, Japan), and protein blocker (Dako, Carpinteria, CA, USA), followed by fixation in 4% PFA. An anti-mouse MBP antibody (BioLegend, San Diego, CA, USA, 808,401, 1:500) and rabbit anti-mouse neurofilament-200 antibody (NF200; Sigma-Aldrich, N4142, 1:1000) were used as primary antibodies at 4 °C overnight. FITC-conjugated donkey anti-mouse IgG (Jackson ImmunoResearch, 715-095-150, 1:100) and TRITC-conjugated goat anti-rabbit IgG (Jackson ImmunoResearch, 711-025-152, 1:100) were used as secondary antibodies following 1 h staining at room temperature and mounted with VECTASHIELD mounting medium with DAPI (Vector Laboratories, Burlingame, CA, USA). Images were acquired using an FV1000-D microscope (Olympus, Tokyo, Japan). For each group, 12 fields were randomly selected and photographed using fluorescence microscopy. For quantification, the myelination index (MI) was calculated using the following formula: MI = MBP-NF200 colocalization area/NF200-stained area. NF200-stained and MBP-NF200 colocalization areas were measured using ImageJ software.

### Primary cultures

Mixed glial cells were harvested from cerebral cortices of postanal day 3–4 B6 mouse pups. The cerebellum and meninges were carefully removed, and the cerebral cortices were cut into small pieces and digested with 0.25% trypsin at 37 °C for 7 min. DNase I was added at a final concentration of 10 mg/mL for another 8 min. After neutralizing trypsin with fetal calf serum (FCS), samples were centrifuged at 400 × g. Mixed glial cells were then resuspended in glial medium [Dulbecco’s Modified Eagle’s Medium with High Glucose (Gibco; Thermo Fisher Scientific) supplemented with 10% FCS (Gibco) and 1% penicillin/streptomycin] and filtered with a 70 μm cell strainer (Falcon; Corning, New York, USA). Cell density was adjusted to 2 × 10^6^/mL and cells were seeded in poly-L lysine (PLL; Sigma-Aldrich) pre-coated T75 flasks (Corning), for culture with glial medium for 7 d.

### OPC sorting, culture, and immunoglobulin treatment

OPC sorting was performed on day 7 of primary culture with a mouse CD140a (platelet-derived growth factor subunit α, PDGFRα) MicroBead Kit (Miltenyi, Bergisch Gladbach, Germany) and LS columns (Miltenyi) according to the manufacturer’s protocol. OPCs were cultured in PLL pre-coated chamber slides (Sigma-Aldrich) at a density of 2 × 10^5^ cells/300 μL of glial medium supplemented with 2% B27 (Gibco) and IgA, IgM, or IgG at a final concentration of 10 μg/mL for 7 d. Cells were fixed with 4% PFA for 45 min then permeabilized and blocked with PBS containing 1% Triton X, 2% bovine serum albumin, and protein blocker for 30 min at room temperature. Rabbit anti-mouse Olig2 (Abcam, Cambridge, UK, ab109186, 1:600) and rat anti-mouse MBP (Merck, MAB386, 1:500) were used as the primary antibodies for staining at 4 °C overnight. TRITC-conjugated donkey anti-rabbit IgG (Jackson ImmunoResearch, 711-025-152, 1:100) and Alexa Fluor 488-conjugated donkey anti-rat IgG (Jackson ImmunoResearch, 712-545-153, 1:100) were used as secondary antibodies for 1 h staining at room temperature. Subsequently, slides were mounted with VECTASHIELD mounting medium and stored at 4 °C in the dark. Images were captured with a BZ-X700 fluorescence microscope, and myelinated areas were measured using ImageJ software.

### Proliferation and nanofiber assay

To assay proliferation, OPCs were cultured in glial medium containing immunoglobulin and 10 ng/mL PDGF⍺ (PeproTech, Cranbury, NJ, USA) and basic fibroblast growth factor (FGF-basic) (PeproTech) for 24 h. BrdU (Sigma-Aldrich) was then given at a final concentration of 50 mM for another 3 h until cells were fixed.

The nanofiber assay was performed by placing nanofiber plate inserts (Nanofiber Solution, Dublin, OH, USA) into 12-well plates pre-coated with 5 μg/mL PLL overnight. After three PBS washes, 2 × 10^5^ OPCs were seeded and cultured in glial medium supplemented with 2% B27 medium and IgA, IgM, or IgG at a final concentration of 10 μg/mL for 7 d. Cell sample fixation, permeabilization, and blocking were performed with protocols described above. Rabbit anti-mouse Olig2 antibody (Abcam, ab109186, 1:600), rat anti-mouse MBP antibody (Merck, MAB386, 1:500), and rat anti-BrdU antibody (Abcam, ab6326, 1:500) were used as primary antibodies at 4 °C overnight. TRITC-conjugated donkey anti-rabbit IgG (Jackson ImmunoResearch, 711-025-152, 1:100) and Alexa Fluor 488-conjugated donkey anti-rat IgG (Jackson ImmunoResearch, 712-545-153, 1:100) were used as secondary antibodies following 1 h staining at room temperature. Slides were mounted with VECTASHIELD mounting medium and stored at 4 °C in the dark. Images of myelinated fibers were captured with an FV1000-D microscope (Olympus). BrdU-stained areas were imaged with a BZ-X700 fluorescence microscope. All images were quantified using ImageJ software. In the nanofiber assay, number and length of myelin sheaths of at least 50 cells were measured in each group.

### Immunofluorescence staining of Fcγ receptors and IgG subclass interaction assay

OPCs were isolated and cultured according to the protocol described above. For immunofluorescence staining of Fcγ receptors, rat anti-mouse FcγRI (R&D Systems, Minneapolis, MN, USA, MAB20741, 1:500) and goat anti-mouse FcγRIII (R&D Systems, AF1960, 1:500) were used as primary antibodies for staining at 4 °C overnight. Alexa Fluor 488-conjugated donkey anti-goat (Jackson ImmunoResearch, 705-545-003, 1:100) and Alexa Fluor 488-conjugated donkey anti-rat (Jackson ImmunoResearch, 712-545-153, 1:100) were used as secondary antibodies for 1 h staining at room temperature before mounting slides with VECTASHIELD medium. For the IgG subclass interaction assay, purified mouse IgG1, IgG2a, IgG2b, and IgG3 (BioLegend) were added to OPC cultures at a final concentration of 10 μg/mL for 7 d. Subsequently, immunofluorescence staining was performed according to the protocols described above. Images of Fcγ Receptors were captured with an FV1000-D microscope. IgG subclass interaction assay images were captured with a BZ-X700 fluorescence microscope and measured using ImageJ software.

### Reverse transcription PCR (RT-PCR)

Total RNA was collected from isolated OPCs, splenocytes, bone marrow cells and liver mononuclear cells with an RNeasy mini kit (Qiagen, Hilden, Germany). Using ReverTra Ace qPCR RT Master Mix (Toyobo, Osaka, Japan), cDNA was synthesized from 500 ng of RNA. RT-PCR was performed with a 7500 Fast Real-Time PCR System (Applied Biosystems, Foster City, CA, USA) with Fast SYBR Green Master Mix (Thermo Fisher Scientific). PCR products were analyzed with 2% agarose gel electrophoresis and electrophoresis bands were stained with GelRed (Biotium, San Francisco, CA, USA). Glyceraldehyde-3-phosphate dehydrogenase (GAPDH) was used as loading control. The specific primers used in this study are as follows: Gapdh sense, 5′-GGTTGTCTCCTGCGACTTCA-3′; Gapdh antisense, 5′-GCCGTATTCATTGTCATACCAGG-3′; Fcgr1 sense, 5′-GGACAGTGGCGAATACAGGT-3′; Fcgr1 antisense, 5′-GAGTAGCAGCCAATCATTGTGG-3′; Fcgr2b sense, 5′-ACCCTTCCAGAGGAAGTAGGT-3′; Fcgr2b antisense, 5′-ATCAGGAGGATTGTATGGGCTG-3′; Fcgr3 sense, 5′-CTGCTGCTGTTTGCTTTTGC-3′; Fcgr3 antisense, 5′-TTCGCACATCAGTGTCACCA-3′; Fcgr4 sense, 5′-GGGTTCCGGATATCTGTGGTG-3′; Fcgr4 antisense, 5′-ACAGCCTTTTGGAGACCAGC-3′.

### Statistical analysis

Statistical analyses were performed using GraphPad Prism v8.1.2 (GraphPad Software, San Diego, CA, USA). Comparisons between groups were performed using a one-way analysis of variance followed by post hoc Tukey's multiple comparisons test. *p* < 0.05 was set as the significance level.

## Results

### Exacerbation of cuprizone-induced demyelination in B cell-deficient mice

**Figure 1:**
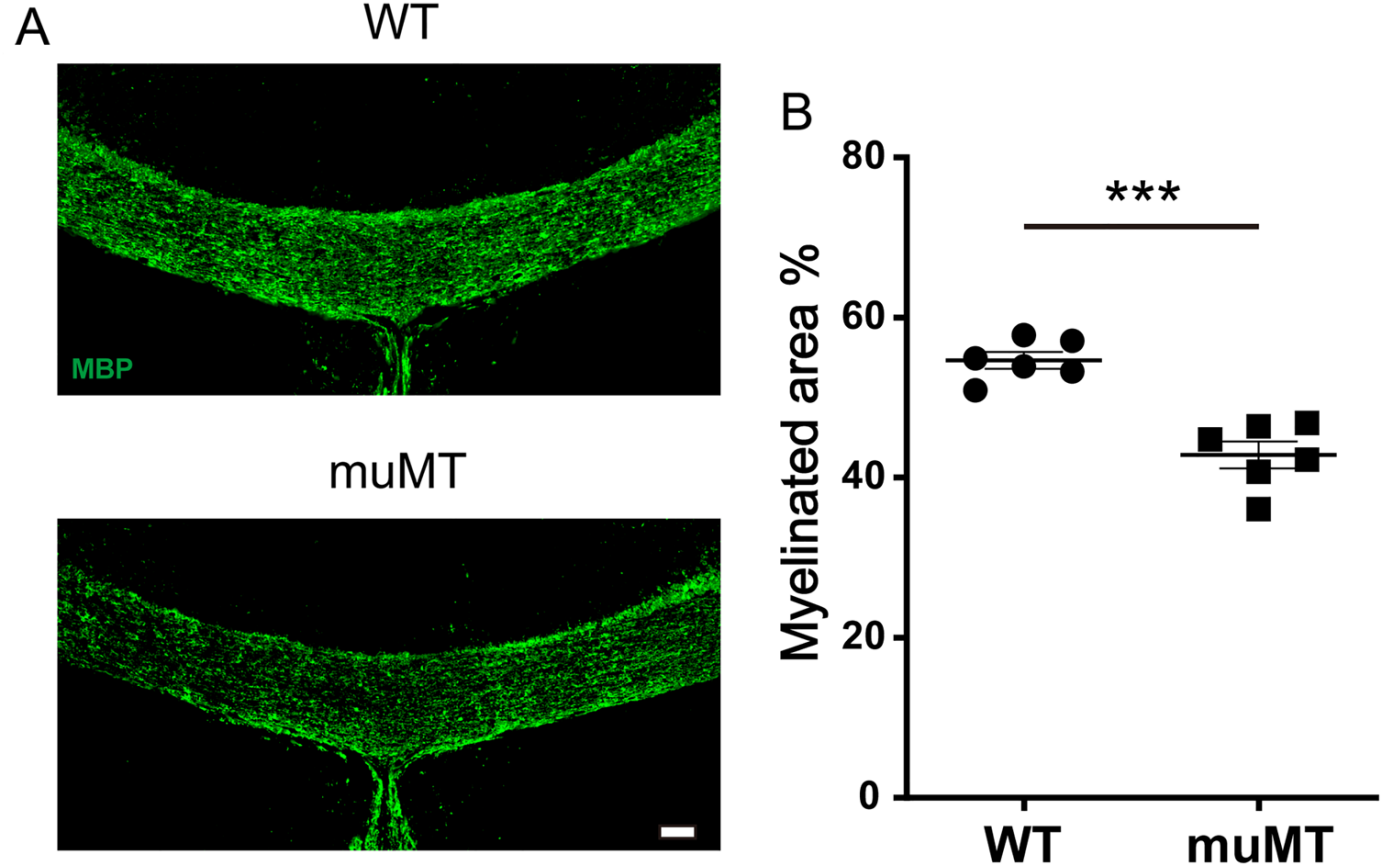
Exacerbation of cuprizone-induced demyelination in B cell-deficient mice. (**A**) MBP staining of the corpus callosum from B cell-deficient mice and WT mice. Scale bar, 100 μm. (**B**) Myelinated area of the corpus callosum (n = 3 mice per group, pooled from two independent experiments). Data are expressed as the mean ± SEM. Unpaired t test was used for statistical analysis. ***p < 0.001.

To analyze the effect of B cells on demyelination, we induced a cuprizone demyelination model in B cell-deficient mice and their wild-type (WT) littermates. Three weeks after the start of cuprizone administration, brains were removed from mice and immunohistochemically stained for MBP to measure the degree of demyelination. We confirmed that there is no difference in the myelinated area of the corpus callosum at Bregma 0.0 mm between cuprizone-naïve muMT mice and WT littermates (Supplementary Fig. S1). Analysis of the corpus callosum at Bregma 0.0 mm from cuprizone-treated mice revealed that the myelinated area was significantly smaller in B cell-deficient mice compared with WT mice, indicating that demyelination was exacerbated (Fig. 1A,B). Analysis at the Bregma 0.6 mm area also showed that demyelination tended to be worse in B cell-deficient mice, although the difference did not reach statistical significance. This result suggests that B cells have a suppressive role in cuprizone-induced demyelination.

### Immunoglobulin enhanced remyelination following LPC-induced demyelination

**Figure 2:**
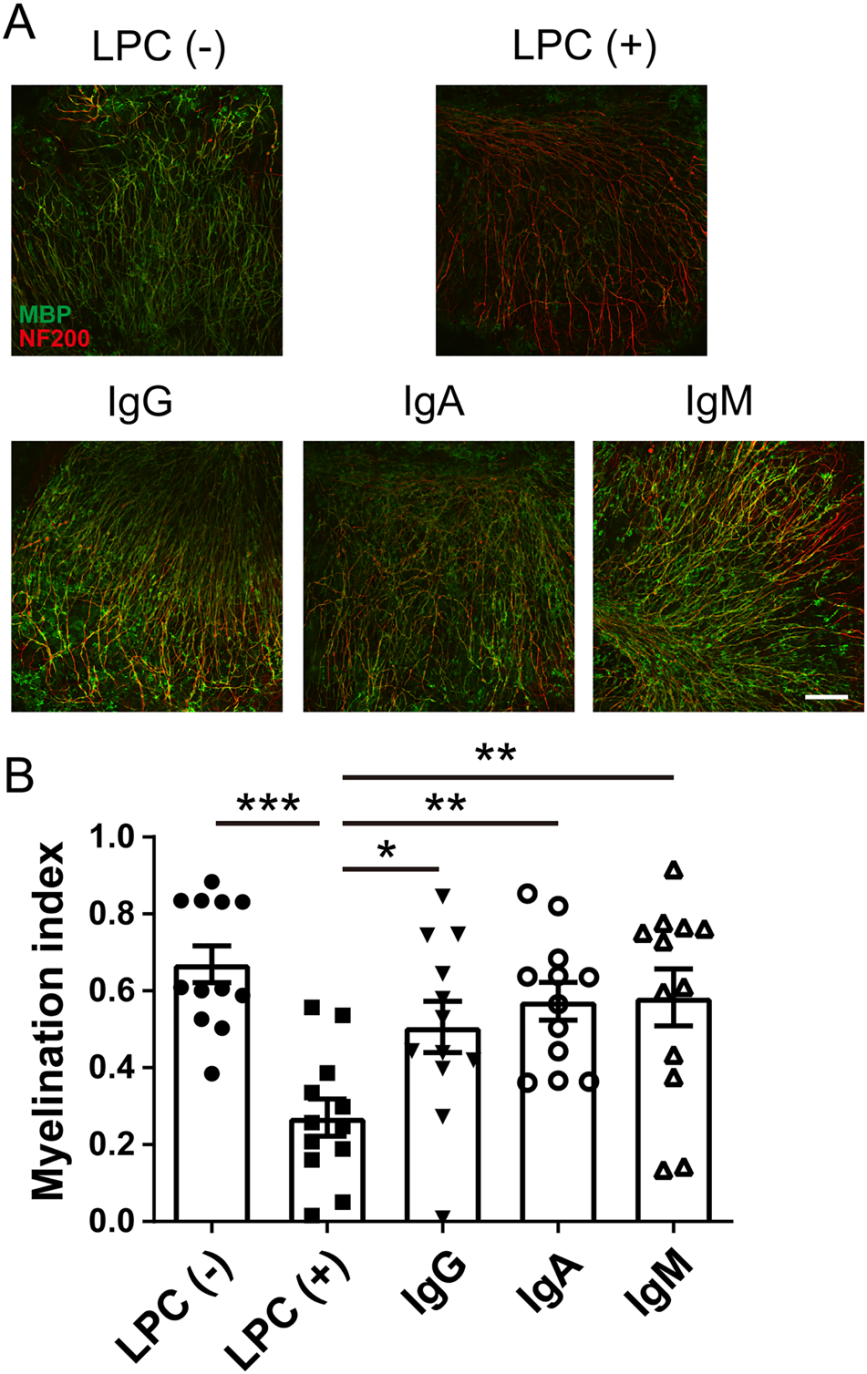
Immunoglobulin-enhanced remyelination following LPC-induced demyelination. (**A**) MBP and NF200 immunocytochemical staining of organotypic brain slice cultures. Scale bar, 100 μm. (**B**) Myelination index of organotypic cerebellar slice cultures (n = 3 slices per group, pooled from four independent experiments). Data are expressed as the mean ± SEM. One-way analysis of variance followed by post hoc Tukey’s multiple comparisons test was used for statistical analysis. *p < 0.05; **p < 0.01; ***p < 0.001.

Because immunoglobulin have been shown to enhance myelination during mouse development, we next investigated whether immunoglobulin affected the myelin formation process. To investigate the involvement of immunoglobulin in myelin formation, we analyzed the effect of immunoglobulin on remyelination following demyelination in vitro using a mouse organotypic brain slice culture system. After 6 d of incubation, demyelination was induced by LPC. IgG, IgA, or IgM were added after 24 h of LPC treatment. After 7 d incubation, immunohistological analysis was performed to evaluate proportions of myelinated axons (Fig. 2A). The LPC-exposed group showed a loss of myelin compared with the control group. Treatment with IgG, IgA, or IgM significantly increased MBP expression, indicating enhanced remyelination following LPC-induced demyelination compared with the LPC alone group (Fig. 2B). This result suggests that immunoglobulin had a facilitative effect on myelination and contributed to remyelination from LPC-induced demyelination in the organotypic brain slice culture system.

### Immunoglobulin promoted OPC differentiation and myelination

**Figure 3:**
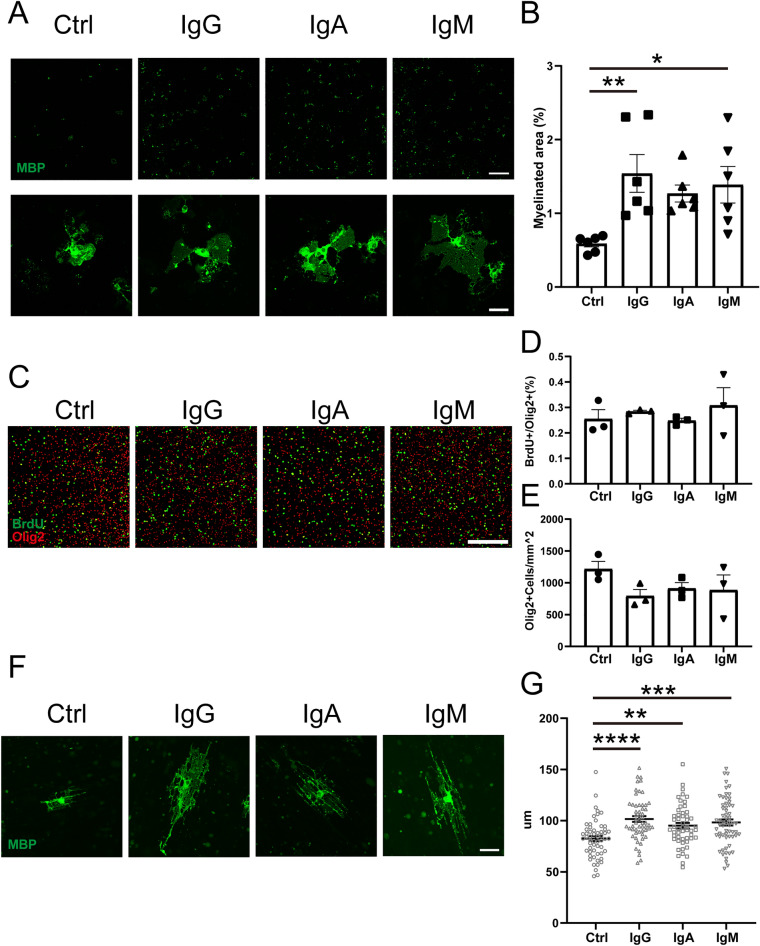
Immunoglobulin-enhanced OPC differentiation and myelination. (**A**) MBP immunocytochemical staining of OPC cultures. Scale bar, 500 μm. The lower row shows magnified figure of OPCs. Scale bar, 50 μm. (**B**) MBP-positive area of OPC cultures (n = 3 cultures per group, pooled from two independent experiments). (**C**) Olig2 and BrdU immunocytochemical staining of OPC cultures. Scale bar, 1000 μm. (**D**) Ratio of BrdU-positive cells among Olig2-positive cells (n = 3 cultures per group). (**E**) Numbers of Olig2-positive cells (n = 3 cultures per group). (**F**) MBP immunocytochemical staining of OPCs with nanofiber cultures. Scale bar, 50 μm. (**G**) Length of myelin sheaths. Data are expressed as the mean ± SEM. One-way analysis of variance followed by post hoc Tukey’s multiple comparisons test was used for statistical analysis. *p < 0.05; **p < 0.01; ***p < 0.001; ****p < 0.0001.

The organotypic brain slice culture system contains not only oligodendrocytes but also various cells such as neurons, astrocytes, and microglia. Therefore, to investigate whether immunoglobulin had a direct effect on OPCs, we analyzed the effect of immunoglobulin using OPC monoculture. We prepared primary mixed glial cultures from P3–5 mice and cultured them for 7 d. OPCs were isolated using anti-CD140a (PDGFRα) beads and cultured in medium supplemented with IgG, IgA, or IgM. After 7 d, immunohistochemical staining was performed to evaluate expression of MBP, a marker of oligodendrocyte maturation, by measuring the area of positive staining. Compared with the control group, MBP-positive areas were significantly larger in the IgG, IgA, and IgM groups, indicating that the addition of immunoglobulin promoted differentiation of OPCs into oligodendrocytes (Fig. 3A,B). These results suggest that immunoglobulin may act directly on OPCs to promote their differentiation.

We first addressed the effect of immunoglobulin on OPC by adding BrdU to OPC cultures and measuring the ratio of BrdU-positive cells among Olig2-positive cells. Compared with the control group, there were no significant differences in ratios of BrdU-positive cells among Olig2-positive cells in IgG-, IgA-, or IgM-treated groups (Fig. 3C,D). Numbers of Olig2-positive cells were also not significantly different between control and immunoglobulin-treated groups (Fig. 3E), indicating that immunoglobulins did not affect OPC proliferation.

Although immunoglobulin did not affect OPC proliferation, it increased MBP expression, suggesting that immunoglobulin promoted OPC differentiation. As another indicator of differentiation, we examined the effect of immunoglobulin on myelination of nanofibers by cultured OPCs. The length of myelin sheaths was significantly longer in IgG-, IgA-, and IgM-treated groups compared with the control group (Fig. 3F,G), although the number of myelin sheaths was not significantly different between groups. These results suggest that immunoglobulins act directly on OPCs to promote their differentiation and myelination.

### OPCs express FcγRI and FcγRIII

**Figure 4:**
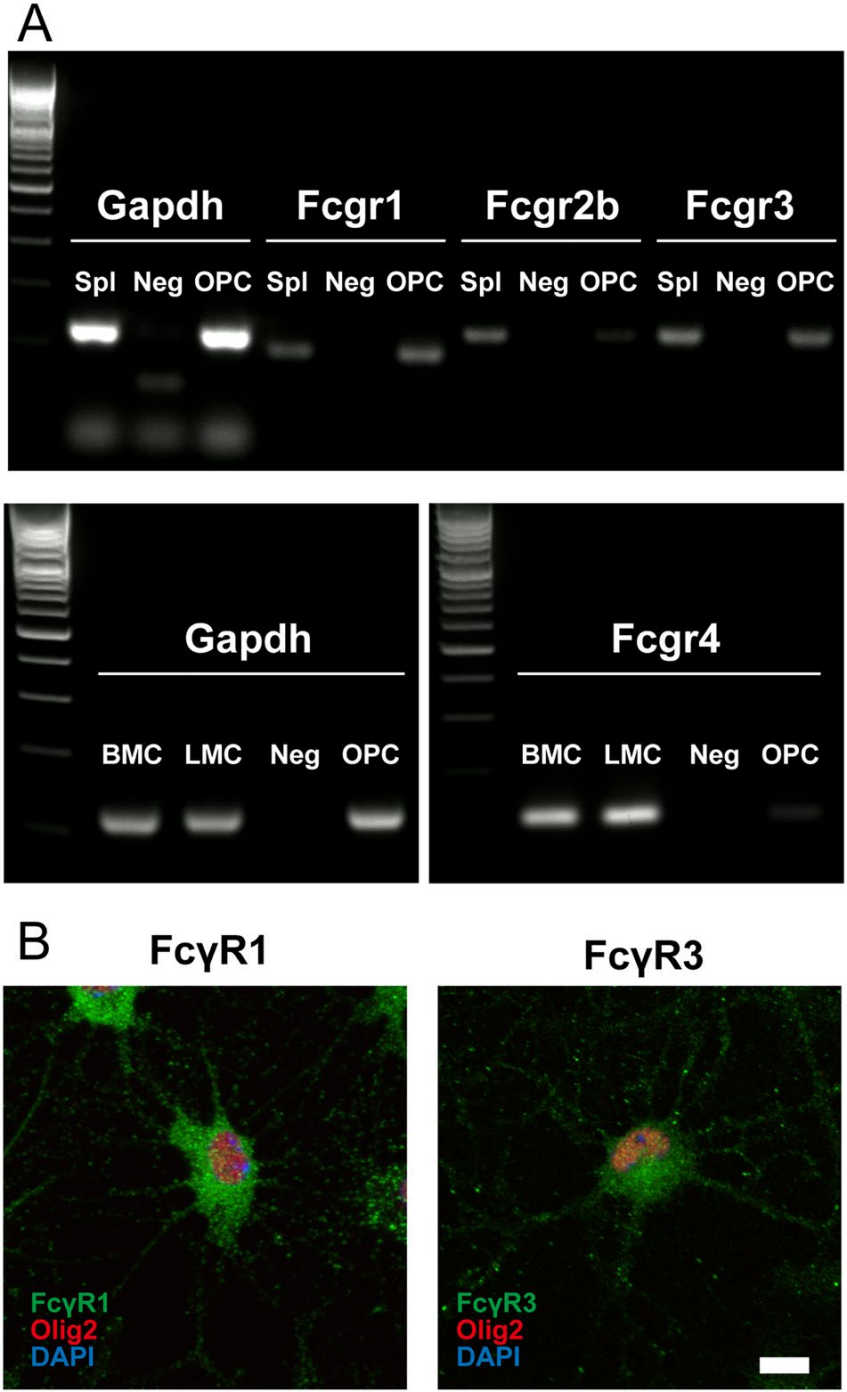
OPCs express FcγRI and FcγRIII. (**A**) Expression analysis of FcγRs in OPC cultures with RT-PCR. Spl, Splenocyte; Neg, Negative control; BMC, Bone marrow cells; LMC, Liver mononuclear cells. (**B**) FcγRI or FcγRIII immunocytochemical staining of OPC cultures. Scale bar, 10 μm. Data are expressed as the mean ± SEM.

Next, we analyzed the expression of receptors for immunoglobulin Fc to clarify the mechanism by which immunoglobulins act directly on OPCs. Regarding Fc receptor expression in mouse OPCs, FcamR, a receptor for IgM and IgA, is expressed on OPCs^18^. Although the FcR gamma chain (FcRγ) is also expressed on OPCs and involved in their differentiation^20^, the expression of individual FcγRs has not been studied in detail. Thus, we analyzed the expression of the four currently known FcγRs (FcγRI, FcγRIIb, FcγRIII, and FcγRIV) using RT-PCR. We found that FcγRI and FcγRIII were expressed on OPC (Fig. 4A). Further analysis using immunofluorescent staining revealed that FcγRI and FcγRIII were expressed on OPCs (Fig. 4B). These results suggest that IgG may act through these two receptors.

### Effects of each IgG subclass on OPCs

**Figure 5:**
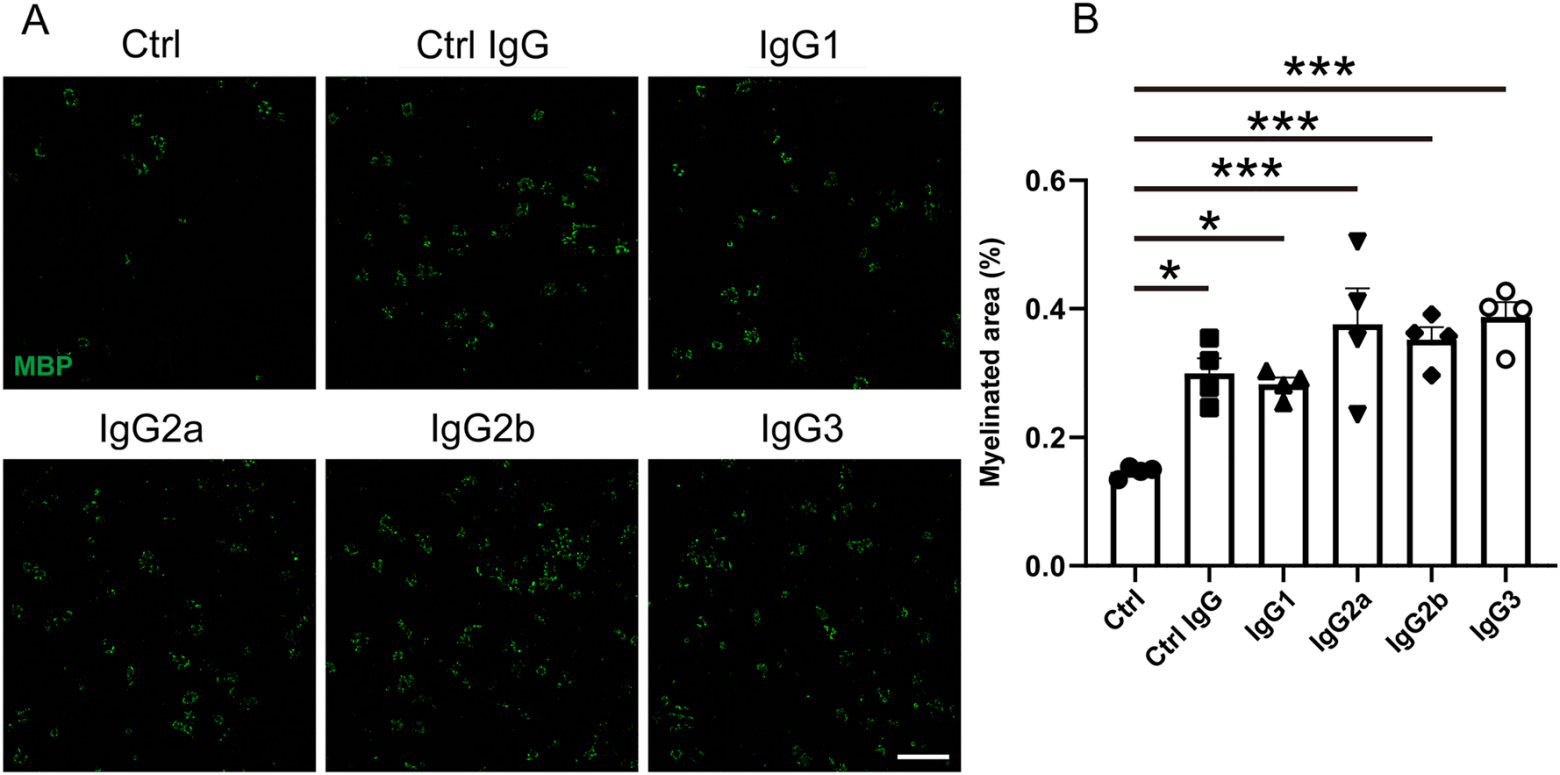
IgG1, IgG2a, IgG2b, and IgG3 enhance OPC differentiation. (**A**) MBP immunocytochemical staining of OPC cultures. Scale bar, 500 μm. (**B**) MBP-positive area of OPC cultures (n = 4 cultures per group). Data are expressed as the mean ± SEM. One-way analysis of variance followed by post hoc Tukey’s multiple comparisons test was used for statistical analysis. *p < 0.05; **p < 0.01; ***p < 0.001.

To further analyze which receptors for IgG are involved, we investigated the effect of each subclass of IgG on OPC differentiation. There are five subclasses of mouse IgG: IgG1, IgG2a, IgG2b, IgG2c, and IgG3. FcγRI shows high affinity to IgG2a, IgG2b, and IgG3, but no affinity to IgG1. In contrast, FcγRIII has affinity to IgG1, IgG2a, and IgG2b, but no affinity to IgG3^21^. We cultured OPCs in medium supplemented with each IgG subclass for 7 d and then performed immunohistochemical staining to evaluate MBP expression by measuring positively stained areas. We found that the MBP-positive area was significantly larger in each IgG subclass-treated group compared with the control (Fig. 5A,B). Furthermore, there were no statistically significant differences in MBP-positive areas among the subclasses. These results indicate that the effect of IgG on OPCs is mediated by both FcγRI and FcγRIII receptors.

## Discussion

In this study, we showed that B cell deficiency exacerbates cuprizone-induced demyelination. Moreover, we revealed that immunoglobulins promote remyelination following LPC-induced demyelination, and immunoglobulins act directly on OPCs to promote their differentiation. We also found that the action of IgG on OPCs is mediated by two receptors, FcγRI and FcγRIII.

Cuprizone, which was used to induce demyelination in mice, is a copper chelator. Oral administration of cuprizone induces mitochondrial dysfunction due to copper deficiency. As a result, oligodendrocyte metabolism and function are impaired, and demyelination is induced in the white matter of the brain. However, the detailed mechanism of why oligodendrocytes are selectively impaired is still unclear^22^. The involvement of B cells in a cuprizone-induced demyelination model has not previously been reported. However, studies with recombination activating gene (RAG)-knockout mice showed that cuprizone-induced demyelination in mice lacking T cells and B cells did not differ from that of control mice, suggesting that T cells and B cells may not be involved in cuprizone-induced demyelination^22–24^. However, our results indicate that B cell deficiency exacerbated cuprizone-induced demyelination. The cause of this discrepancy between our results and those of RAG-deficient mice is unclear. However, mice lacking interleukin (IL)-17, a cytokine produced by T cells, or the receptor for IFN-γ showed mitigation of cuprizone-induced demyelination^22^. Therefore, it is possible that the adverse effects of B cell deficiency on demyelination antagonized the inhibitory effect of the lack of T cell-derived cytokine production on demyelination in RAG-knockout mice, resulting in no difference from control mice. In this study, we evaluated the demyelination at 3 weeks after the start of cuprizone administration. In the cuprizone-induced demyelination model, it is known that demyelination is completed approximately 5 weeks after the start of cuprizone administration, and remyelination is observed approximately 2 weeks after discontinuation of administration^25^. We attempted to evaluate demyelination and remyelination at later time points. However, at 5 weeks, the corpus callosum were highly demyelinated, and it was difficult to detect any differences between muMT mice and WT littermates. Furthermore, the subsequent remyelination process was highly variable and difficult to assess in our study. We therefore analyzed the remyelination using in vitro culture systems.

We next investigated the effect of immunoglobulins on remyelination using an organotypic brain slice culture system and found that immunoglobulins promote remyelination following LPC-induced demyelination. Previous studies reported that OPCs express FcRγ and its deficiency inhibits myelination during mouse brain development^20^. B cell-derived IgM has a promotive role in myelination during mouse development^18^. It has been reported that naturally occurring autoantibodies that recognize CNS autoantigens promote remyelination^26,27^. In particular, rHIgM22, which is a recombinant form of human IgM naturally occurring autoantibody, has been shown to stimulate OPC proliferation in vitro, and promote remyelination in Theiler’s virus infection-induced and curpizone-induced demyelination in vivo^26,27^. The Fc receptor-mediated effect of immunoglobulin may also contribute to increased remyelination following demyelination. In patients with MS, the remyelination process is reportedly impaired^28^. Their findings and ours suggest that immunoglobulins play a promotive role in remyelination, potentially leading to the development of new therapeutic strategies for demyelinating diseases such as MS.

Recently, the role of B cells in the pathogenesis of MS has attracted attention due to the clinical application of anti-CD20 antibodies, which reportedly have a strong relapse-suppression effect in RRMS patients, making them an important treatment option^13,29^. Administration of anti-CD20 antibodies rapidly depletes CD20^+^ B cells from the peripheral blood. However, immunoglobulin-producing plasma cells and plasmablasts, which do not express CD20, are not directly affected by anti-CD20 antibodies. In addition, because the effect of anti-CD20 treatment is exerted early, even before decreases in concentrations of IgM and IgG in the serum occur^13^, the IgG concentration, IgG index, and number of OCB in the CSF do not change before and after administration of anti-CD20 antibody^30^. Accordingly, the therapeutic effect of anti-CD20 antibodies is unlikely to be mediated by a reduction of IgG concentration. Instead, anti-CD20 antibodies are thought to exert their therapeutic effect by suppressing B cell production of cytokines, such as tumor necrosis factor α, IL-6, and granulocyte–macrophage colony-stimulating factor^31–33^, and chemokines such as CXCL13 and CCL19^34^; and inhibiting the function of B cells as antigen-presenting cells to T cells^35^. OCB has been detected in the spinal fluid of many MS patients, indicating intrathecal immunoglobulin production occurs. However, it has also been reported that OCB recognizes constitutively expressed intracellular antigens and may be produced secondarily to tissue destruction^12^. These findings suggest that the role of autoantibodies in the pathogenesis of MS is unclear and immunoglobulins do not necessarily have a promotive effect on MS pathogenesis. Furthermore, a recent study using experimental autoimmune encephalomyelopathy (EAE), an animal model of MS, reported that IgA^+^ plasma cells were dramatically reduced in the gut and accessed the CNS during EAE to suppress disease severity^36^. Moreover, gut microbiota-specific IgA-producing cells reportedly infiltrate active inflammatory lesions in human MS patients^37^. Although the role of these IgA-producing cells remains unclear, IL-10-mediated actions have been suggested. Our results indicate that IgA produced by these cells may contribute to remyelination.

Intravenous immunoglobulin (IVIg) is also used in the treatment of autoimmune diseases^38^. However, a double-blind study of patients with RRMS revealed that IVIg was not effective for endpoints such as relapse frequency and novel magnetic resonance imaging lesions. Nevertheless, IVIg was reportedly effective in early clinical trials^39–42^. Furthermore, a combination of biomarkers could distinguish responders from non-responder patients^43^, further obscuring the effect of IVIg in MS. The mechanism by which IVIg is effective is thought to mainly occur through its effects on the peripheral immune system, such as inhibiting activation of macrophages, dendritic cells, and pathogenic T cells; suppressing inflammatory cytokine production; inhibiting pathogenic autoantibodies; and suppressing complement activation via anti-idiotypes^38,44^. The intrathecal effects of IVIg are thought to be limited because of its low transition into the CSF. Thus, uncertainty about the therapeutic effect of IVIg in MS does not exclude the possibility of the beneficial effect of immunoglobulins on OPC differentiation and remyelination in humans.

In our studies using the OPC monoculture system, the addition of immunoglobulin resulted in significant increases in MBP-positive areas. Furthermore, nanofiber analysis indicated a significant increase in myelin length in the immunoglobulin group. The addition of immunoglobulin did not affect OPC proliferation but may have a direct effect on OPC differentiation and myelination. OPCs reportedly express Fcα/μR, and IgM promotes OPC differentiation and myelination through Fcα/μR during mouse brain development^18,45^. IgG is known to promote OPC differentiation via FcRγ^20^, but it was unknown which IgG Fc receptor actually mediates these effects. Our study revealed that OPCs express FcγRI and FcγRIII, and IgG acts through both FcγRI and FcγRIII on OPCs, indicating that all subclasses of IgG can affect OPCs.

Our findings reveal that the presence of B cells has an inhibitory effect on cuprizone-induced demyelination. We also show that immunoglobulins enhanced remyelination following LPC-induced demyelination in an organotypic brain slice culture system. Our results indicate that immunoglobulin directly affected OPCs and promoted their differentiation and myelination, and that this action was mediated by both FcγRI and FcγRIII receptors. Further analysis of the detailed mechanisms underlying the effects of immunoglobulins on OPCs, as well as their role in vivo, will lead to development of novel treatments for MS and other demyelinating diseases.

### Supplementary Information

Supplementary Information.

## Data Availability

The datasets used and analyzed during the current study are available from the corresponding author on reasonable request.
