# Author Correction: Immunoglobulin directly enhances differentiation of oligodendrocyte-precursor cells and remyelination

**DOI:** 10.1038/s41598-023-40118-4

**Published:** 2023-08-10

**Authors:** Yaguang Li, Daisuke Noto, Yasunobu Hoshino, Miho Mizuno, Soichiro Yoshikawa, Sachiko Miyake

**Affiliations:** 1 Department of Immunology, Juntendo University School of Medicine, 2-1-1 Hongo, Bunkyo-ku, Tokyo, 113-8421 Japan; 2 Department of Neurology, Juntendo University School of Medicine, Tokyo, Japan

Correction to: *Scientific Reports* 10.1038/s41598-023-36532-3, published online 09 June 2023

The original version of this Article contained an error in the Discussion section.

“However, to our knowledge, the effect of immunoglobulin on remyelination following demyelination has not been reported. In patients with MS, the remyelination process is reportedly impaired^26^. Our results suggest that immunoglobulins play a promotive role in remyelination, potentially leading to the development of new therapeutic strategies for demyelinating diseases such as MS.”

now reads:

“It has been reported that naturally occurring autoantibodies that recognize CNS autoantigens promote remyelination^26,27^. In particular, rHIgM22, which is a recombinant form of human IgM naturally occurring autoantibody, has been shown to stimulate OPC proliferation in vitro, and promote remyelination in Theiler’s virus infection-induced and curpizone-induced demyelination in vivo^26,27^. The Fc receptor-mediated effect of immunoglobulin may also contribute to increased remyelination following demyelination. In patients with MS, the remyelination process is reportedly impaired^28^. Their findings and ours suggest that immunoglobulins play a promotive role in remyelination, potentially leading to the development of new therapeutic strategies for demyelinating diseases such as MS.”

As a result of this error, References 26 and 27 were added and are listed below.

26. Watzlawik, J. O., Wootla, B., Painter, M. M., Warrington, A. E. & Rodriguez, M. Cellular targets and mechanistic strategies of remyelination-promoting IgMs as part of the naturally occurring autoantibody repertoire. *Expert Rev. Neurother.* **13**, 1017–1029 (2013).

27. Fereidan-Esfahani, M., Nayfeh, T., Warrington, A., Howe, C. L. & Rodriguez, M. IgM natural autoantibodies in physiology and the treatment of disease. In *Methods in Molecular Biology* vol. 1904 (ed. Steinitz, M.) 53–81 (Humana Press, New York, 2019).

The subsequent References have been renumbered in the text and the Reference list.

The original Article has been corrected.

