# Supplementary material for: Immunoglobulin directly enhances differentiation of oligodendrocyte-precursor cells and remyelination
Source: Sci Rep. 2023 Jun 9;13:9394. doi: 10.1038/s41598-023-36532-3 (PMC10256778; doi:10.1038/s41598-023-36532-3)
Supplement: Supplementary file 1 — Supplementary Information. [file 41598_2023_36532_MOESM1_ESM.pdf]

Supplementary information

Immunoglobulin directly enhances differentiation of oligodendrocyte-precursor cells and remyelination

Yaguang Li[1], Daisuke Noto[1], Yasunobu Hoshino[2], Miho Mizuno[1], Soichiro Yoshikawa[1], Sachiko Miyake[1*]

[1]Department of Immunology, Juntendo University School of Medicine, Tokyo, Japan

[2]Department of Neurology, Juntendo University School of Medicine, Tokyo, Japan

Corresponding author

Sachiko Miyake, MD, PhD

Department of Immunology, Juntendo University School of Medicine, 2-1-1 Hongo, Bunkyo-ku, Tokyo, 113-8421, Japan
Tel.: +81 (33) 813-1044
Fax: + 81 (33) 813-0421
e-mail: s-miyake@juntendo.ac.jp
ORCID: 0000-0001-6349-2936

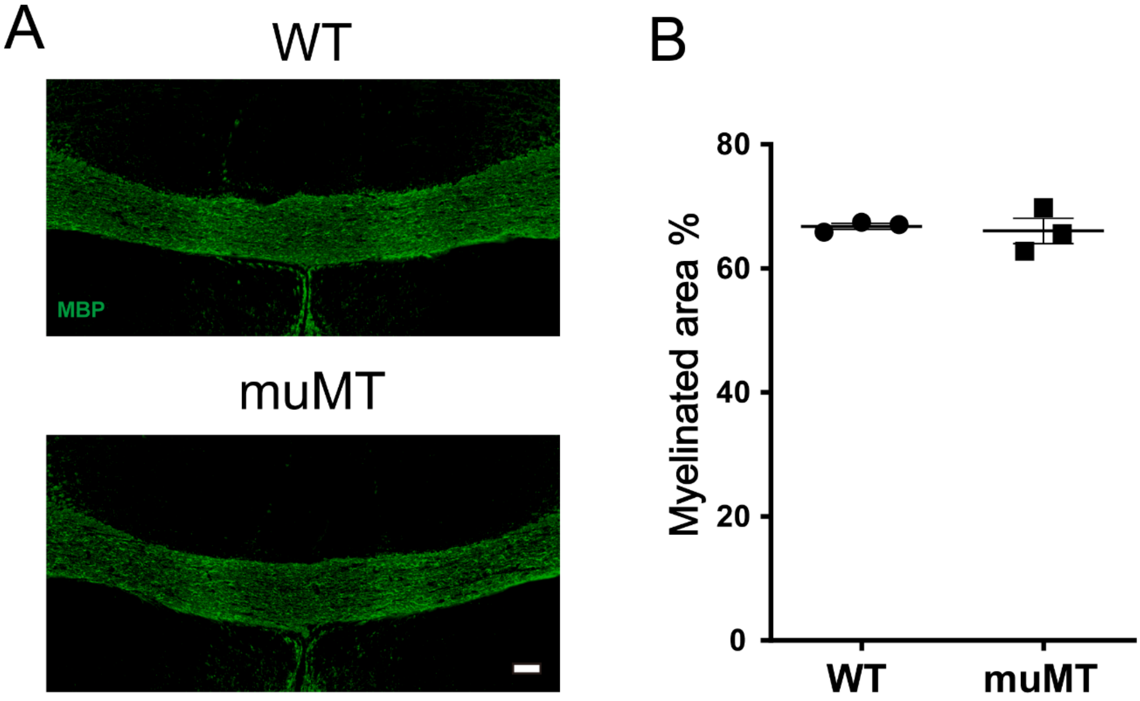

Supplementary Fig. S1. Myelination area of cuprizone-naïve mice.

(a) MBP staining of the corpus callosum from B cell-deficient mice and WT mice. Scale bar, 100 μm. (b) Myelinated area of the corpus callosum (n = 3 mice per group). Data are expressed as the mean ± SEM.
